# Myositis induced by durvalumab in a patient with non‐small cell lung cancer: A case report

**DOI:** 10.1111/1759-7714.13709

**Published:** 2020-10-26

**Authors:** Miyuki Kobayashi, Masafumi Saiki, Chisa Omori, Shuichiro Ide, Kazuki Masuda, Yusuke Sogami, Takanori Hata, Hiroshi Ishihara

**Affiliations:** ^1^ The Department of Internal Medicine II, Faculty of Medicine University of Yamanashi Chuo Japan; ^2^ The Department of Neurology University of Yamanashi, Faculty of Medicine Chuo Japan

**Keywords:** Durvalumab, non‐small cell lung cancer, immune‐related adverse events, myositis

## Abstract

Immune checkpoint inhibition is associated with a broad spectrum of immune toxicities referred to as immune‐related adverse events (irAEs). Myositis is known to be a potentially fatal irAE. Here, we report a case of immune‐related myositis after the administration of durvalumab. A 60‐year‐old man with stage IIIA lung adenocarcinoma was treated with durvalumab after concurrent chemoradiation therapy. After the third dose of durvalumab, his serum CK level was elevated, and soon thereafter myalgia of the proximal muscles and blepharoptosis were observed. We diagnosed immune‐related myositis based on the results of pathological examination and initiated systemic corticosteroid therapy. His symptoms then improved and the serum CK level immediately dropped to within a normal range. Clinicians should be aware of possible myositis during the early phase of durvalumab therapy.

## Graphical abstract

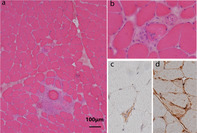

We report a case of immune‐related myositis after administration of durvalumab in patient with non‐small cell lung cancer (NSCLC).

## Introduction

Durvalumab is a human monoclonal antibody against programmed cell death ligand 1 (PD‐L1). It is approved for maintenance therapy after definitive chemoradiation therapy in unresectable locally advanced non‐small cell lung cancer (NSCLC). Immune checkpoint inhibitors (ICIs) exhibit marked therapeutic effects but are also associated with inflammatory side effects related to increased immune activity in the form of immune‐related adverse events (irAEs). Although rare, myositis and rhabdomyolysis have been reported as fatal irAEs. Here, we report a case of myositis after the third dose of durvalumab in a patient with lung adenocarcinoma.

## Case report

A 60‐year‐old man who was a past smoker with a Brinkman index of 1080, underlying dilated cardiomyopathy and type 2 diabetes, was diagnosed with unresectable locally advanced lung adenocarcinoma (cT2bN2M0‐stage IIIA) in January 2018. Molecular analyses revealed that the tumor was negative for epidermal growth factor receptor mutations and anaplastic lymphoma kinase gene rearrangements and that 1%–24% of the tumor cells expressed PD‐L1.

**Figure 1:**
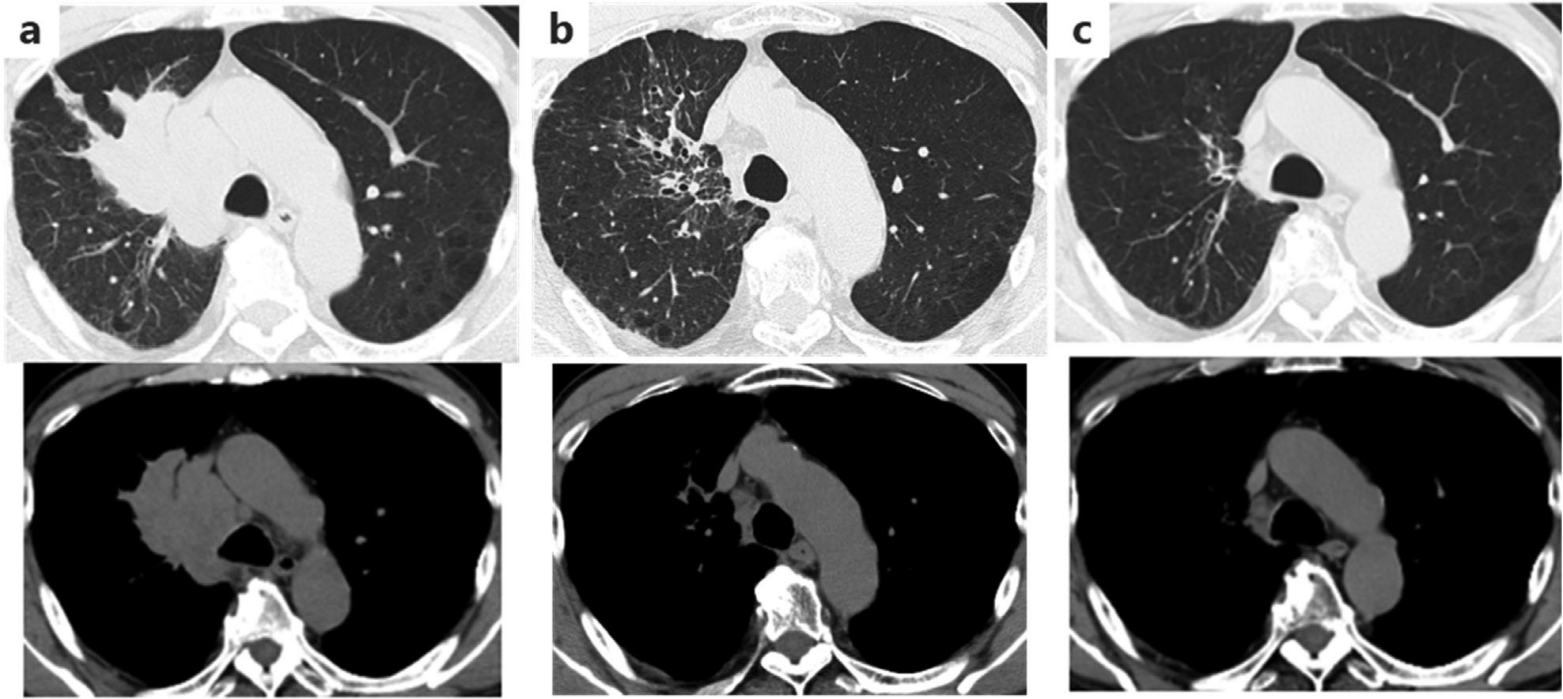
Chest computed tomography (CT) images (**a**) at diagnosis; (**b**) before administration of durvalumab; and (**c**) at the onset of immune‐related myositis after the third course of durvalumab treatment when the size of the lung mass had decreased dramatically.

He was treated with chemotherapy (weekly carboplatin and paclitaxel) and concurrent radiotherapy. When chemoradiotherapy was completed, chest computed tomography (CT) revealed a partial response (Fig 1). Durvalumab as consolidation therapy was started seven weeks after the first chemotherapy day. After the third dose of durvalumab, laboratory testing revealed elevated serum creatine phosphokinase (CK) (1317 U/L; normal range (NR) <187 U/L), but he was asymptomatic. Although durvalumab treatment was discontinued, the following week he presented with myalgia of the proximal muscles and blepharoptosis, and his serum CK was further elevated (3278 U/L).

**Figure 2:**
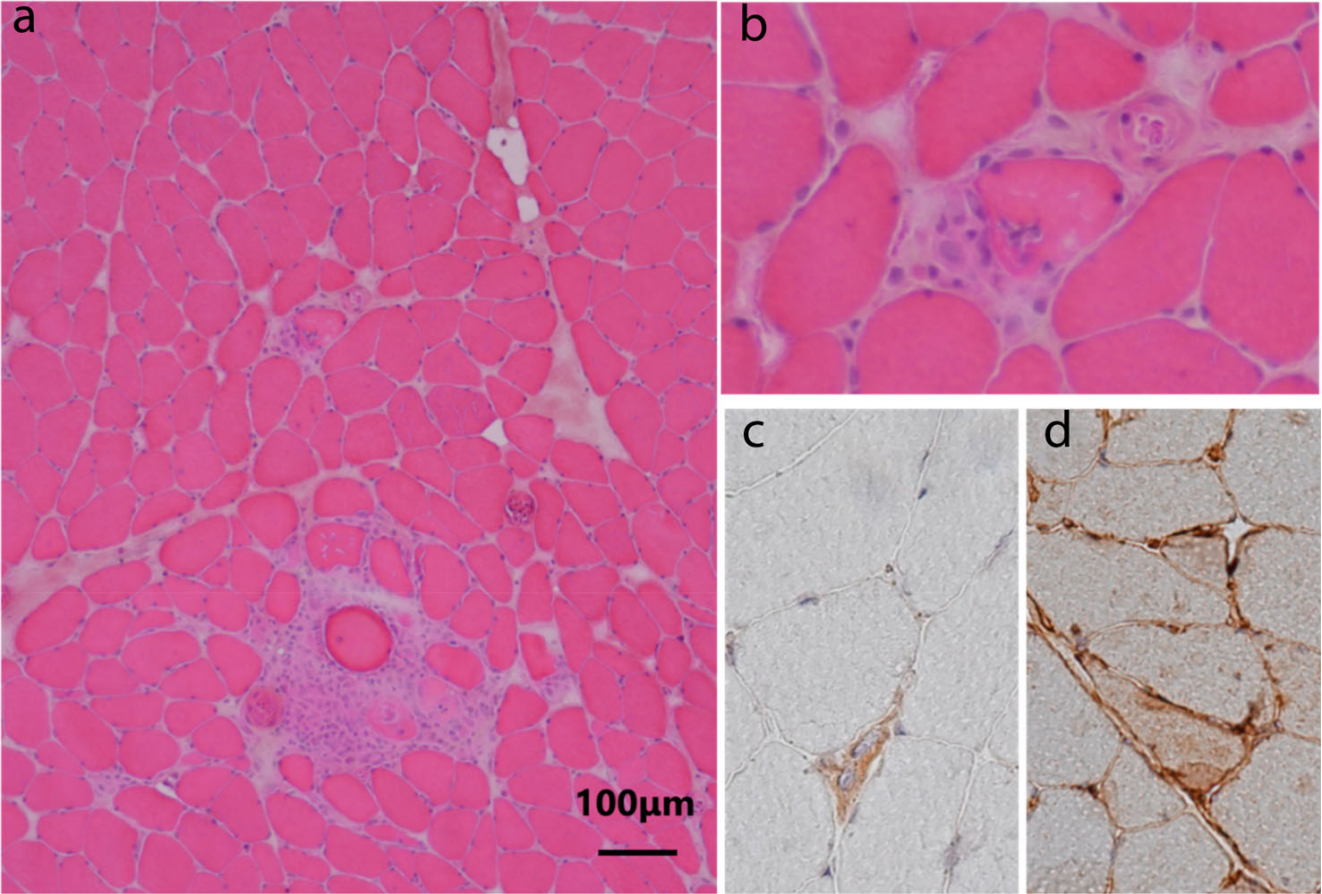
Histopathological features in skeletal muscle (right quadriceps). (**a**, **b**) Hematoxylin‐eosin staining showed slight variation in diameter of muscle fibers. Necrotic fibers and infiltration of mononuclear cells into the endomysium are visible. (**c**) CD8‐positive T lymphocytes. (**d**) Major histocompatibility complex class 1 immunohistochemical stained the sarcolemma of myofibers.

On physical examination, the patient was noted to have myalgia in the neck, shoulder blade, thigh, and blepharoptosis. No muscle weakness of the limbs or sensory impairment was observed. His vital signs were stable. Laboratory tests revealed elevated levels of aldolase (22.9 IU/L; NR <5.9 IU/L), and lactate dehydrogenase (581 IU/L; NR < 472IU/l). Myositis‐specific autoantibodies including anti‐ARS, anti‐MDA5, anti‐TiF1γ, anti‐Mi2, anti‐Ku and myasthenia gravis‐related antibodies including anti‐AChR and anti‐MuSK were negative. CK‐MB was elevated (30.7 ng/mL; NR < 6.3 ng/mL) but less than 10% of CK. Neither an electrocardiogram nor 2‐D echocardiogram revealed myocardiopathy. Needle electromyogram of the right quadriceps femoris muscle revealed myogenic changes with low‐amplitude and short‐duration motor unit potentials and an early recruitment pattern. Repetitive nerve stimulation test of the right median nerve did not show a waning phenomenon. Edrophonium and ice‐pack tests did not show clear improvement of ptosis. Muscle biopsy of the right quadriceps femoris (Fig 2) showed small groups of necrotic fibers scattered over a wide area. Endomysial infiltration of mononuclear cells were observed. However, cellular inflammatory infiltrates with invasion of non‐necrotic muscle fibers, a prerequisite for polymyositis diagnosis, was absent, as was perifascicular atrophy, characteristic of dermatomyositis. A small number of CD8‐positive cells was confirmed by immunostaining, and major histocompatibility complex (MHC) class I‐positive fibers were observed. The patient was finally diagnosed with immune‐related myositis (irMyositis) due to durvalumab.

**Figure 3:**
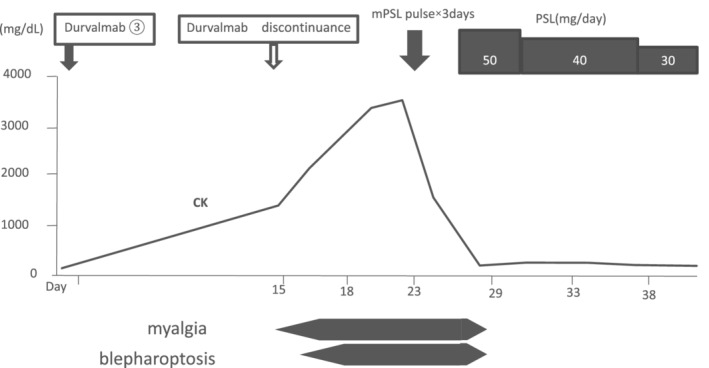
After three courses of durvalumab, the creatine kinase (CK) level was 3433 U/L (normal range < 187 U/L). Soon after administration of three days of steroid pulse therapy (methylprednisolone 1000 mg/day), the patient's subjective symptoms (myalgia and blepharoptosis) showed improvement, and CK levels quickly normalized. Prednisolone 50 mg/day was started and gradually tapered, but no relapse of symptoms or CK elevation was observed.

Soon after administration of 1000 mg methylprednisolone daily for three days, subjective symptoms began to improve, and serum CK levels quickly normalized. Prednisolone 50 mg daily was started and tapered, but no relapse of symptoms or CK elevation was observed (Fig 3). We considered resuming durvalumab treatment, but did not do so at the patient's request. Nonetheless, he did not show apparent signs of relapse of lung cancer until one year after discontinuation of durvalumab.

## Discussion

PD‐L1 is expressed in antigen‐presenting cells and is thought to negatively regulate the immune response by binding to PD‐1 expressed in activated lymphocytes.^1^ PD‐L1 is also expressed in tumors, and these malignancies may exploit the PD‐1/PD‐L1 pathway to attenuate antitumor immunity.^2^ Durvalumab blocks PD‐L1 binding to PD‐1 and CD80, allowing T cells to recognize and kill tumor cells. Although ICIs can lead to significant clinical benefit, they are also associated with irAEs. In skeletal muscle, activation of PD‐1/PD‐L1 signaling has been shown to regulate the immune response and maintain tolerance to self‐antigens,^3^ and inhibition of PD‐1/PD‐L1 signaling by durvalumab may enhance T cell activity of normal muscle tissue against self‐antigens, resulting in irMyositis.

The skeletal muscle pathology of irMyositis includes infiltration of mononuclear cells, especially CD8+ T cells, into muscle fibers.^4^ Additionally, it features upregulated MHC class I expression and increased PD‐L1 expression on muscle fibers and mononuclear cells. All of these are consistent with the mechanism of irMyositis.^5^ Similar findings from the skeletal muscle biopsy of this case support a diagnosis of irMyositis.

The estimated frequency of IrMyositis is 0.58%–0.76% among all PD‐1 inhibitor‐treated patients,^6^ and its incidence with durvalumab monotherapy was 0.2% in the PACIFIC study.^7^ This indicates that the combination of radiation therapy and ICIs does not increase the risk of irMyositis. IrMyositis develops in the early phase after treatment initiation (2 to 15 weeks, median 3 weeks), and progresses rapidly after onset. The risk factors for irMyositis in particular are unknown; however, as for other irAEs, the pre‐existence of autoimmune‐mediated conditions (such as anti‐ARS syndrome) is a risk factor for flare‐ups of pre‐existing autoimmune disease or the development of irAEs.^4^ Clinical manifestations are dominated by myalgia, limb‐girdle weakness, axial weakness, ptosis, and oculomotor weakness with diplopia; myocarditis and respiratory muscle paralysis are major causes of death.^5^ Although irMyositis is potentially fatal, reports on the clinical course of irMyositis are limited, especially with regard to patients treated with durvalumab.

In summary, here we describe a case of irMyositis that developed after the third dose of durvalumab therapy as consolidation treatment for lung adenocarcinoma. Serum CK level was elevated before clinical symptoms, allowing early diagnosis and therapeutic intervention. The patient recovered completely from irMyositis with systemic corticosteroid therapy. His clinical symptoms had progressed rapidly, and if diagnosis and treatment had been delayed, his symptoms might have become more severe and a fatal outcome could have ensued as a complication of myocarditis and respiratory muscle paralysis. Clinicians therefore should be aware of the possibility of potentially fatal irMyositis during the early phase of durvalumab therapy.

## Disclosure

No authors report any conflict of interest.
